# Patients undergoing subacute rehabilitation have accurate expectations of their health-related quality of life at discharge

**DOI:** 10.1186/1477-7525-10-94

**Published:** 2012-08-17

**Authors:** Steven McPhail, Terry Haines

**Affiliations:** 1 Centre for Functioning and Health Research, Buranda Plaza, Corner Ipswich Road and Cornwall Street, Buranda, Brisbane, Australia; 2 Institute of Health and Biomedical Innovation and School of Public Health & Social Work, Queensland University of Technology, Kelvin Grove, Brisbane, Australia; 3 Southern Health, Allied Health Research Unit, Kingston Centre, Cnr Warrigal and Kingston Roads, Cheltenham, Victoria, Australia; 4 Physiotherapy Department, School of Primary Health Care, Monash University, Monash University Peninsular Campus, Victoria, Australia

## Abstract

**Background:**

Expectations held by patients and health professionals may affect treatment choices and participation (by both patients and health professionals) in therapeutic interventions in contemporary patient-centered healthcare environments. If patients in rehabilitation settings overestimate their discharge health-related quality of life, they may become despondent as their progress falls short of their expectations. On the other hand, underestimating their discharge health-related quality of life may lead to a lack of motivation to participate in therapies if they do not perceive likely benefit. There is a scarcity of empirical evidence evaluating whether patients’ expectations of future health states are accurate. The purpose of this study is to evaluate the accuracy with which older patients admitted for subacute in-hospital rehabilitation can anticipate their discharge health-related quality of life.

**Methods:**

A prospective longitudinal cohort investigation of agreement between patients’ anticipated discharge health-related quality of life (as reported on the EQ-5D instrument at admission to a rehabilitation unit) and their actual self-reported health-related quality of life at the time of discharge from this unit was undertaken. The mini-mental state examination was used as an indicator of patients’ cognitive ability.

**Results:**

Overall, 232(85%) patients had all assessment data completed and were included in analysis. Kappa scores ranged from 0.42-0.68 across the five EQ-5D domains and two patient cognition groups. The percentage of exact correct matches within each domain ranged from 69% to 85% across domains and cognition groups. Overall 40% of participants in each cognition group correctly anticipated all of their self-reported discharge EQ-5D domain responses.

**Conclusions:**

Patients admitted for subacute in-hospital rehabilitation were able to anticipate their discharge health-related quality of life on the EQ-5D instrument with a moderate level of accuracy. This finding adds to the foundational empirical work supporting joint treatment decision making and patient-centered models of care during rehabilitation following acute illness or injury. Accurate patient expectations of the impact of treatment (or disease progression) on future health-related related quality of life is likely to allow patients and health professionals to successfully target interventions to priority areas where meaningful gains can be achieved.

## Background

Expectations held by patients and health professionals may affect treatment choices and participation (by both patients and health professionals) in therapeutic interventions in contemporary patient-centered healthcare environments [1,2]. Accurate understanding of likely therapeutic benefit and the personal capacity for improvement is constructive for informing joint decision making between patients and health professionals and to facilitate adherence to therapeutic protocols [2,3]. This is particularly important amongst patients who have suffered a severe health event where the primary goal of intervention is rehabilitation intended to maximize health-related quality of life rather than providing a simple intervention for curative effect [4,5]. This is the case for hospitalised older adults; a high priority clinical group who consume large amounts of healthcare resources [6].

Subacute in-hospital rehabilitation amongst older adults following acute illness or injury is one clinical setting where issues pertaining to health-related quality of life are paramount [7,8]. The nature of health conditions causing older adults to be admitted to hospital often necessitates intensive periods of hospital rehabilitation; including multiple therapies [8,9]. Patient-centered models of care are becoming increasingly popular in rehabilitation settings [10-16]. In order for a patient-centered model of care to be employed in subacute hospital rehabilitation settings, patients are required to understand and integrate complex health and treatment related information in order to participate in joint goal setting and planning potential home environment adaptations [2,3]. However it is common for patients to have poor health literacy and for health professionals to underestimate patients’ desire for health information [3,17].

Accurate expectations of the impact of treatment (or disease progression) on future health-related related quality of life is desirable in rehabilitation settings to allow patients and health professionals to target interventions to priority areas where meaningful gains can be achieved [18]. Similarly, achieving functional gains in these areas are likely to contribute to sustained motivation for participating in rehabilitative therapies. On the other hand, if patients’ expectations are too high they may become despondent when their progress does not meet their own outlook. This may lead to loss of motivation and result in less than optimal rehabilitation outcomes. Similarly, sub-optimal outcomes may occur if patients’ expectations are too low. Patients with low expectations may see little reward in continuing to invest effort in therapies they perceive will not result in further improvement to their health-related quality of life. However, there is a currently a scarcity of empirical data to inform dialogue on this topic. To date, there have been no investigations of agreement between patients’ expected and actual health-related quality of life on discharge from inpatient rehabilitation.

This investigation aimed to evaluate the accuracy with which patients admitted for subacute inpatient rehabilitation can anticipate their discharge health-related quality of life as reported on the Euroqol-5D (EQ-5D) instrument.

## Methods

### Design

A prospective longitudinal cohort investigation of agreement between patients’ anticipated discharge health-related quality of life and their actual discharge health-related quality of life was undertaken.

### Participants and setting

Two hundred and seventy-two patients (consecutive admissions) from a subacute geriatric assessment and rehabilitation unit at a tertiary hospital participated in this investigation. Patients admitted to this unit for multi-disciplinary rehabilitation had overcome the acute phase of their injury or illness causing hospitalisation, but required further multidisciplinary rehabilitation to maximize their recovery and health-related quality of life on discharge and thereafter. No specific sample size calculation was carried out prior to commencement due to the novel nature of this observational study (sample size of convenience).

## Materials

To evaluate health-related quality of life the EQ-5D [19] instrument was used. This generic health-related quality of life instrument includes six questions. The first five are three level multiple choice relating to the domains of mobility, personal care, usual activities, pain / discomfort, and anxiety / depression. The simplistic nature of the three level multiple choice domain questions made it particularly suitable for use amongst this older adult population. The three levels for each domain refer to 1. no problems, 2. some problems / moderate and 3. unable / extreme. The three level multiple choice categories were considered appropriate for patients (without specialist medical knowledge or high levels of health literacy) to anticipate their own future health state.

The Dolan tariff system was applied to these responses to produce a multi-attribute utility score (utility) where death and perfect health are represented by 0 and 1 respectively (health states considered worse than death are assigned negative values) [20]. The full range of utility scores that can be derived from this tariff system include values from −0.594 to 1.00 [20]. This tariff system was selected as it was derived from a population with similar cultural and societal attributes to the society from which these patients belong and has been utilised more than any other tariff system in psychometric studies of the EQ-5D instrument relevant to this clinical population [21-27]. The sixth and final question from this instrument is a 100 point health state Visual Analogue Scale (EQ-VAS) where 0 and 100 are represented by worst and best imaginable health respectively.

The EQ-5D has a range of empirical evidence supporting its internal, external, concurrent and construct validity across a wide variety of populations and patient groups; including older adults [22,23,25,26,28-33]. There is also a substantial volume of evidence supporting various aspects of its reliability [22,27,31,32,34]. Empirical investigations have also demonstrated that the EQ-5D has sound sensitivity to change [35-39]. A review of 8 investigations incorporating 11 patient groups revealed a median minimally important difference in health utility from the EQ-5D of approximately 0.08 [40].

Due to the prevalence of cognitive impairment amongst clinical populations of this nature, agreement between anticipated and actual discharge reports of health-related quality of life was examined for the sample as a whole; as well as by level of cognitive ability indicated by the Mini-Mental State Examination (MMSE) score. [41] The Mini-Mental State Examination (MMSE) was used as a broad indicator of patient cognitive ability [41]. The MMSE incorporates a brief assessment of orientation, memory, attention and arithmetic and is routinely completed for all patients in the participating clinical unit [41]. A comprehensive review of empirical evidence for this instrument concluded that it was an appropriate instrument to quantitatively assesses the severity of cognitive impairment [42]. There is evidence supporting its criterion validity, construct validity and reliability, including its suitability for use amongst older adults [42-45].

Patients from this clinical group may (or may not) have cognitive impairment. For the purpose of analysis, each patient from the sample was classified into a better cognition group (admission MMSE greater than 23) or a poorer cognition group (admission MMSE less than or equal to 23) [46]. This cut-off is consistent with prior studies among older adults, despite being somewhat arbitrary in nature [42-46]. The MMSE was completed on admission to the rehabilitation unit, and was repeated again for any patient who the treating clinical team considered may have experienced a change in cognitive ability (e.g. developed acute delirium). However, patients are unlikely to be admitted or discharged from the unit while experiencing acute changes in their cognitive status. Patients who experienced a cognitive event, such as delirium as a result of an infection, received immediate treatment to resolve the acute delirium and were not discharged home until after they had returned to their usual level of cognitive functioning. All participants in this sample were in the same cognition grouping at admission and discharge.

### Procedure

Patients completed a standard battery of clinical assessments conducted by their physical therapists on the first weekday of their admission. In this way, all admission assessments were undertaken within 72 hours of admission to the unit. This assessment included physical performance tests as well as an interview administered EQ-5D for patients to report their health-related quality of life. Patients were then given a blank copy of the EQ-5D and the therapist read the words of the EQ-5D aloud (directly from the EQ-5D text). Patients then marked their response on EQ-5D instrument. The EQ-5D was administered as part of routine assessments for all patients. Physical therapists in this unit received in-service training on how to administer the EQ-5D without influencing patients’ responses by reading directly from the text without leading patients with their tone or non-verbal cues. In addition to this standard training, staff conducting assessments included in this research received one to one instruction from a member of the investigative team to ensure that the EQ-5D was administered without bias at each assessment.

Immediately following the completion of the standard admission EQ-5D, patients were then given a blank EQ-5D questionnaire and a brief scripted statement was read by the clinician conducting the assessment. This scripted statement was to prepare the patients to report their anticipated discharge health-related quality of life (on the EQ-5D instrument). This included a rudimentary outline of what usually occurs during a rehabilitation admission in this hospital unit to provide patients with a consistent frame reference irrespective of which therapist was conducting the assessment. It is also noteworthy that the study did not aim to investigate patients’ ability to predict their length of stay, but rather their discharge health-related quality of life. Therefore the investigators considered the scripted statement necessary for ensuring that all patients had a foundational understanding of what rehabilitation may involve (such as participating in therapies) and knowing approximately how far ahead they were reporting their anticipated quality of life (approximately 42 days in this case). The clinicians were instructed to read this statement at a slow, steady pace for all participants to provide patients with a standard description of what care to expect.

"“You will receive regular therapies and other treatments during your stay to help you get ready for discharge. The average length of stay in this unit is six weeks. Some patients are ready and leave earlier than six weeks. Some patients need to stay longer than six weeks. When you are ready to be discharged from this unit, which of these statements do you think will best describe your health state at that time?”"

When completing their anticipated discharge EQ-5D, the assessing clinician was permitted to answer patient questions regarding the nature of the treatment they would receive during their inpatient stay. Clinicians were also permitted to read the question again for the patient (directly from the script and the EQ-5D questions) without leading or assisting the patient to select a particular response. For this reason both the admission and anticipated EQ-5D was considered to have been interview administered rather than self administered and clinical staff were considered to be unblinded to patient assessments.

The MMSE was completed by hospital occupational therapists or medical staff for each patient admitted to the unit as part of their routine care. Patients’ admission MMSE assessments were completed within 24 hours of EQ-5D completion. This may have occurred before or after the EQ-5D assessment depending on therapy assessment scheduling within the clinical unit. The MMSE results along with other patient demographic variables were collated from the medical history. Immediately prior to discharge, patients again completed the standardised battery of assessments. This assessment also included an EQ-5D evaluation of their current health state at the time of discharge from the rehabilitation unit.

This research investigation was approved by the institutional human ethical review board who waived the need for individual consent (negligible risk and utilising routine assessments); gatekeeper consent for staff participation was attained from the clinical managers in the unit.

### Analysis

Conventional descriptive statistics were used to describe the sample. Tests of hypothesis were used to examine difference between the lower and higher cognition groups in age (unpaired t-test) and length of stay (Mann–Whitney U). For the individual EQ-5D domain scores, levels of agreement between the anticipated discharge and actual discharge EQ-5D responses were calculated using weighted kappa with disagreements of only one level ascribed a 0.5 weighting; bias corrected 95% confidence intervals for kappa scores were calculated using bootstrap resampling (2000 replications of original sample size, stratifying for cognition grouping where appropriate) [47,48]. The number (and percentage) of exact matches for each of the domains were also tabulated per cognition group.

For the summary EQ-5D scores (utility and EQ-VAS), Limits of Agreement (LOA) [49] and intraclass correlation coefficients were calculated separately for patients in each cognition grouping as well as for the total sample. Bland-Altman plots were prepared for the utility index and EQ-VAS. To investigate systematic differences between anticipated and actual discharge health-related quality of life scores (for utility and EQ-VAS) paired t-tests were employed separately for each cognition group as well as for the whole cohort combined.

## Results

**Figure 1:**
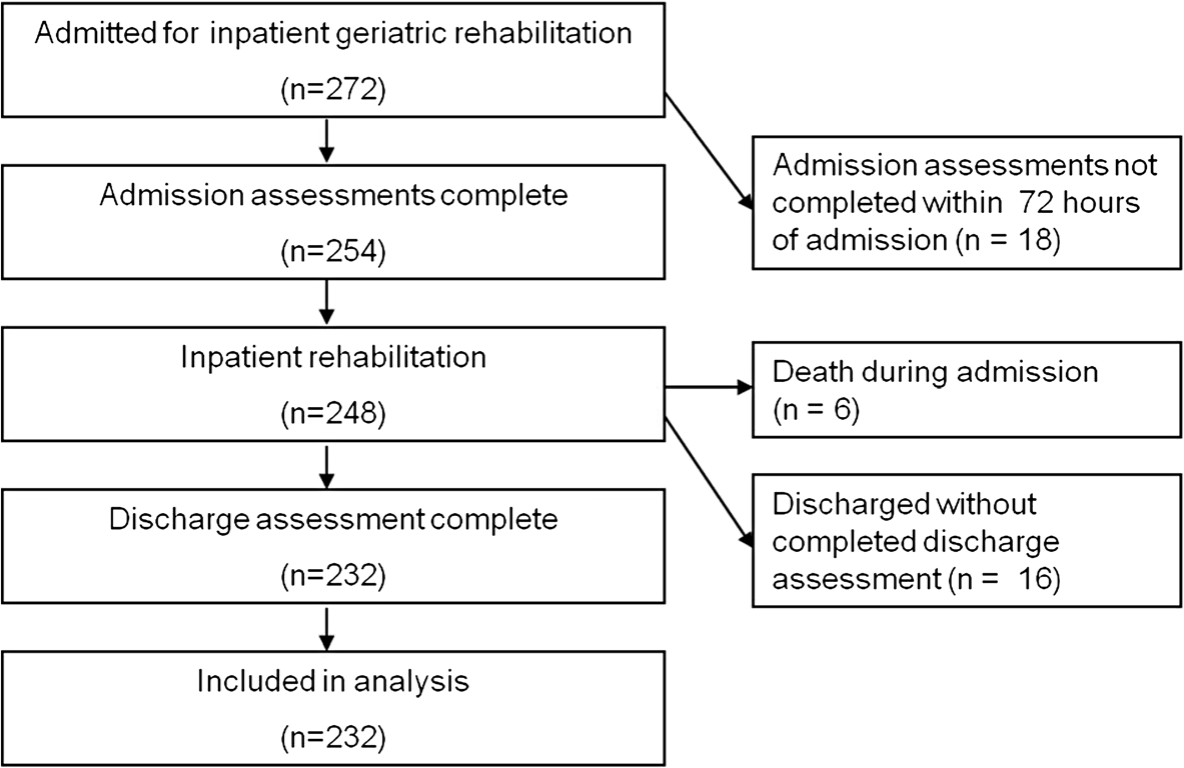
Participant flow diagram for the investigation.

**Figure 2:**
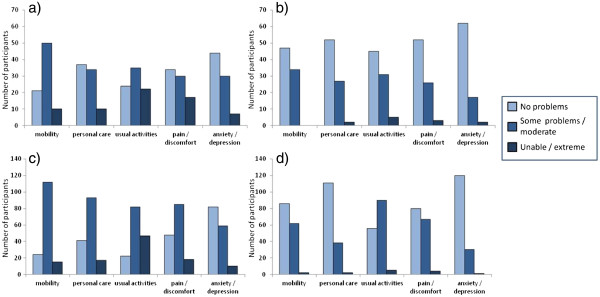
Frequency histograms for the number of participants in each item response category for the lower cognition group at a) admission and b) discharge assessments, as well as for the better cognition group at c) admission and d) discharge.

Participant flow through for the duration of the study is outlined in Figure 1. Overall, 232 (85%) patients had all assessment data completed and were included in analysis. Demographic, primary diagnosis or reason for rehabilitation admission and EQ-5D responses are displayed in Table 1. The lower cognition group was older (p < 0.001) with a mean (SD) age of 79.0 (11.8) in comparison to the better cognition group 71.7 (14.9). The median (IQR) length of stay was 42 (25–66) days; with no difference between cognition groups (p = 0.60). Participants in both cognition groups reported reduced health-related quality of life at admission with scope for improvement (Table 1). A higher proportion of EQ-5D responses at discharge were in the least impaired response categories across the EQ-5D domains (Figure 2).

**Table 1:** Demographics and primary diagnosis (or reason for admission) category for patients included in analysis

	**Better cognition group**	**Poorer cognition group**
	**n = 131**	**n = 81**
Mean Age (standard deviation)	71.7 (14.9).	79.0 (11.8)
Median MMSE (inter-quartile range)	28 (26–30)	20 (17–22)
Female (%)	88 (58%)	51 (63%)
Reason for admission diagnosis category		
Orthopedic	45 (30%)	30 (37%)
Stroke	41 (27%)	12 (15%)
Other Neurological	24 (16%)	11 (14%)
Geriatric re-condition	14 (9%)	12 (15%)
Other disabling condition requiring rehabilitation	27 (18%)	16 (20%)
Admission EQ-5D		
Mean (standard deviation) utility	0.425 (0.352)	0.444 (0.402)
Mean (standard deviation) VAS	57 (19)	63 (19)
Discharge EQ-5D		
Mean (standard deviation) utility	0.748 (0.213)	0.757 (0.264)
Mean (standard deviation) VAS	79 (13)	77 (15)

For agreement between anticipated and actual EQ-5D domain scores, kappa statistics and exact matches are reported in Table 2. Kappa scores ranged from 0.42 to 0.68 across domains and cognition groups. The percentage of exact correct matches within each domain ranged from 69% to 85% across domains and cognition groups. Overall 40% of participants in each cognition group correctly anticipated all of their self-reported discharge EQ-5D domain responses.

**Table 2:** Levels of agreement between anticipated and actual discharge EQ-5D domain responses (kappa and exact guess) for patients undergoing hospital rehabilitation

	**Agreement per domain Kappa (95% CI)**					**Exact match Number (%)**					
	**Mobility**	**Personal Care**	**Usual Activities**	**Pain / Discomfort**	**Anxiety / Depression**	**Mobility**	**Personal Care**	**Usual Activities**	**Pain / Discomfort**	**Anxiety / Depression**	**All domains correct**
Lower cognition (n = 81)	0.59 (0.44,0.71)	0.59 (0.44,0.71)	0.64 (0.50,0.77)	0.58 (0.45,0.70)	0.68 (0.56,0.79)	64 (79%)	66 (81%)	56 (69%)	56 (69%)	62 (77%)	32 (40%)
Better cognition (n = 151)	0.58 (0.39,0.74)	0.58 (0.39,0.74)	0.63 (0.42,0.78)	0.46 (0.27,0.63)	0.42 (0.22,0.61)	119 (79%)	129 (85%)	118 (78%)	127 (84%)	125 (83%)	61 (40%)
Combined (n = 232)	0.58 (0.48,0.68)	0.58 (0.48,0.68)	0.64 (0.53,0.74)	0.55 (0.45,0.65)	0.59 (0.49,0.69)	183 (79%)	195 (84%)	174 (75%)	183 (79%)	187 (81%)	93 (40%)

**Figure 3:**
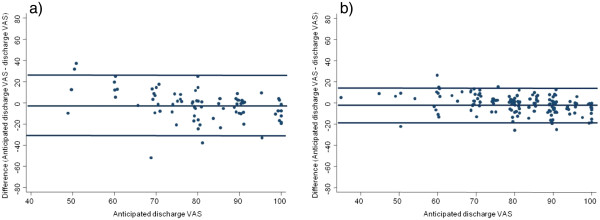
Bland-Altman plots with limits of agreement for difference between anticipated discharge EQ-VAS score and discharge EQ-VAS score for a) participants in the lower cognition group and b) participants in the higher cognition group.

In regard to agreement between anticipated and actual discharge summary scores for the EQ-5D (utility and EQ-VAS), intraclass correlation coefficients and limits of agreement are presented in Table 3. Bland-Altman plots for the EQ-VAS and utility index followed the same pattern (therefore only the EQ-VAS is displayed in Figure 3). The better cognition group had narrower limits of agreement and higher intraclass correlation coefficients than the lower cognition group (Table 3, Figure 3). No mean difference between anticipated and actual discharge utility scores was observed for either cognition group or when both groups were combined. The mean anticipated EQ-VAS was higher than the actual discharge EQ-VAS for the better cognition group (1.9 points, p = 0.010), for both groups combined (2.3 points, p = 0.002), but for the lower cognition group (with a smaller sample size and greater variability in responses) this was not statistically significant at an alpha of 0.05 (3 points, p = 0.063).

**Table 3:** Intraclass correlation coefficient (ICC), mean EQ-5D utility and Visual Analogue Scale (VAS), and limits of agreement (LOA) between anticipated and actual discharge health-related quality of life reports (n = 232)

	**Measure**	**ICC**	**Anticipated mean **	**Actual mean **	**Limits of agreement**			p-value*
		**(95% CI)**	**(95% CI)**	**(95% CI)**	**Lower LOA**	**Mean difference**	**Upper LOA**	
					**(95% CI)**	**(95% CI)**	**(95% CI)**	
Lower cognition	EQ-5D utility	0.72	0.747	0.757	−0.506	0.009	0.524	0.744
		(0.56, 0. 82)	(0.684, 0.811)	(0.698, 0.815)	(−0.563, -0.448)	(−0.048, 0.067)	(0.467, 0.582)	
	EQ-5D VAS	0.63	80.5	77.5	−31.3	−3.0	25.3	0.063
		(0.43,0.76)	(77.6,83.4)	(74.3, 80.8)	(−34.4,-28.1)	(−6.1,0.2)	(22.2, 28.4)	
Better cognition	EQ-5D utility	0.85	0.764	0.748	−0.332	−0.016	0.300	0.211
		(0.79, 0.89)	(0.728, 0.800)	(0.714, 0.782)	(−0.358, -0.307)	(−0.104, 0.091)	(0.274,0.325)	
	EQ-5D VAS	0.86	80.9	79.0	−19.3	−1.9	15.5	0.010*
		(0.81,0.90)	(78.8, 83.0)	(77.0, 81.1)	(−20.7, -17.9)	(−3.3, -0.4)	(14.1, 17.0)	
Combined	EQ-5D utility	0.79	0.758	0.751	−0.405	−0.007	0.390	0.576
		(0.73, 0.84)	(0.726, 0.790)	(0.721, 0.781)	(−0.431, -0.379)	(−0.033, 0.019)	(0.364, 0.416)	
	EQ-5D VAS	0.78	80.8	78.5	−24.1	−2.3	19.6	0.002*
		(0.71,0.83)	(79.1,82.4)	(76.8, 80.2)	(−25.5, -22.7)	(−3.7, -0.8)	(18.1, 21.0)	

Note: *a p-value < 0.05 would indicate that a systematic difference exists (i.e. anticipated discharge health-related quality of life was consistently higher or lower than the actual report at discharge).

## Discussion

Findings from this investigation indicate that patients admitted for inpatient hospital rehabilitation were able to predict their discharge health-related quality of life on the EQ-5D instrument with a moderate level of accuracy in each of the five broad domains. Patients’ health-related quality of life improved in all domains over the duration of their stay. Patients did not systematically overestimate or underestimate their discharge utility score derived from the individual domain responses. However, there was greater variability between anticipated and actual discharge summary scores for the poorer cognition group than the better cognition group. The small observed mean difference in EQ-VAS (2.3 points mean overestimation on the 100 point scale) is unlikely to represent a clinically meaningful difference [50-52].

There was no clear pattern of difference in predicting individual item responses across the individual health-related quality of life domains between the poorer and better cognition groups. The domain kappa scores and exact matches were comparable across cognition groupings and across domains. This may be attributable in part to the limited response options at discharge; where most respondents utilised only the two higher response options. However, the wider LOA among the lower cognition group for the EQ-5D utility index and EQ-VAS indicated that patients in the better cognition group had a smaller error margin than their peers in the lower cognition category.

Comparisons to previous research are difficult given the scarcity of empirical evidence on this topic. This research provides the first empirical evidence indicating that patients undergoing in-hospital rehabilitation have, at worst, moderately accurate expectations of their discharge health-related quality of life. This adds to the weight of foundational evidence supporting joint goal setting and patient centered models of care in rehabilitation contexts for older adults [53-56]. Patients who are well informed about prognosis, the impact of treatment and their future health-related quality of life are more likely to make informed treatment choices to target priority areas where meaningful improvements can be made [53-56]. This may also facilitate participation in therapies and adherence to treatment protocols [53-58]. In contrast, patients who overestimate their discharge health-related quality of life may become anxious, depressed or lose motivation as they fall short of their expectations.

An important consideration when interpreting implications from this study’s findings is that reports of patients’ anticipated health-related quality of life may act as a self-fulfilling prophecy. Those patients who felt helpless and anticipated poor levels of physical functioning, pain and depression may have been less likely to participate in therapies and other treatments [57]. Similarly, patients with a positive outlook and high levels of self-efficacy may have maximized their rehabilitation outcome through active participation during their rehabilitation stay [58]. However, it is not possible to draw strong conclusions in this regard from this observational study design as the degree to which this postulation was true amongst this sample remains uncertain.

Including a comparison between health-professional expectations and patient expectations of discharge health-related quality of life may be a worthwhile undertaking as a future research direction. The notion of patient expectations acting as a self-fulfilling prophecy would be supported if patients who anticipated a poorer outcome then their therapists, did actually achieve a poorer outcome in comparison to those where the patient and health professionals were in agreement. However, investigation of the influence of health-professional expectations on their patients’ expectation for discharge health-related quality of life would also be worthy of consideration. It is plausible that health professional expectations may act as a self fulfilling prophecy if patients considered to have greater potential to improve were provided with additional therapies, treatments or other resources.

It is also possible that patients in this study anticipated the level of functioning that would be required to be discharged safely back into the community and simply reported how they anticipated their health-related quality of life would be if they were to able to function at that level. They may have taken this heuristic response approach by surmising they would not be discharged until that level of functioning had been achieved [59]. This would not necessary have been an undesirable outcome or changed the implications of these findings for patient centered models of care in rehabilitation settings where a common goal of hospital rehabilitation is to prepare patients’ for discharge. Accurate expectations held by patients regarding the level of functioning required for discharge may allow patients and health professionals to target interventions to priority areas of functioning required for successful community living.

Another factor worthy of consideration is whether patients recalled their anticipated discharge health-related quality of life responses and intentionally repeated the same responses at the discharge assessment. The investigators do not believe this occurred for four primary reasons. First, patients completed a wide range of routine assessments from multiple health professional disciplines during the first 72 hours of their admission to the participating rehabilitation unit. The large number of items assessed in this period offered natural protection against recalling their response to the six specific anticipated EQ-5D items. Second, some level of cognitive impairment is present among many patients in this older clinical group. This is evident in the MMSE scores, which indicated a large proportion of patients (including those in the ‘better’ cognition group) were likely to have some difficulty with memory and other rudimentary cognitive functions. Third, the long length of time between assessments (median 6 week length of stay) also provided natural protection against recalling responses from the initial assessment. Fourth, prior research has indicated that patients from comparable clinical groups do not give much consideration to health state scales when reporting their health-related quality of life and do not accurately recall responses to health-related quality of life reports completed at earlier assessments [4,5,60].

A number of caveats should be considered when interpreting findings from this investigation. The EQ-5D is a straightforward instrument with limited response options. In this study the 3-level multiple choice EQ-5D was used. This was a logical choice of instrument for this style of investigation where the objective was to examine a patient reported outcome capturing generic health-related quality of life information. Nonetheless correct prediction of the broad response categories did not require a detailed understanding of their discharge health state (e.g. no problems versus some problems walking around). This is likely to have contributed to a higher level of agreement than that which may have been observed if a more detailed prediction was required. It is also noteworthy that alternative instruments with different psychometric properties may have resulted in more (or less) accurate predictions depending on the qualities of the instrument (response options, sensitivity to change etc.).

There are several factors limiting the extent to which these findings can be generalized. First, all participants were from a single tertiary hospital. Patients from other hospitals or geographical locations may not have responded in the same way. Second, a single generic health-related quality of life instrument was used. Additionally, patients beginning the subacute rehabilitation phase of their recovery are likely to have already been provided with substantial information and advice about their prognosis. Patients in acute hospital care or community based settings may not have the same level of accuracy in anticipating their future health-related quality of life as the sample in this investigation.

A priority for future research following this investigation includes examining patients’ expectations across the continuum of care. This could potentially reveal valuable information regarding the role and timing of health education in joint decision making and patient-centered models of care. The nature of health information and focus of advice is likely to contrast across acute, subacute and community settings. This investigation has also exposed several opportunities for methodological improvement when undertaking future investigations of this nature among older adults. These opportunities include collecting a wider range of patient demographic clinical information that may influence ability to predict future health states. This may include recording patients’ level of education, evaluating patient depression or anxiety levels, determining the amount and content of health education already delivered to patients prior to study commencement and a potential comparison to health professionals accuracy in predicting patients’ future health-related quality of life.

On a broader note, it would also be valuable for future investigations to consider how positive or negative findings regarding patients’ preferences and expectations for their recovery should impact models of service delivery and individual treatment choices. There are many complex ethical considerations that could arise from this line of enquiry. For example, how should health-professionals with a duty of care to their patients respond if inaccurate patient expectations of disease progression (or potential recovery) result in a declination of evidenced based treatments to pursue an unadvisable course of action? How would this response differ depending on the potential severity of outcome or impact on third party dependents, such as children? Many issues in this sphere may initially seem straight forward in the context of patients being central decision makers in their care. Similarly, additional ethical complexity may be exposed if health-professionals do not have some degree of accuracy in anticipating future health-states. To this end, future research should investigate whether health professionals have the ability to predict patients’ future health-related quality of life in a variety of contexts, given that patients are likely to formulate their own expectations after taking into account the opinion of their treating health professionals. Expectations held by health-professionals are likely to directly influence therapies and other treatment options offered to patients.

## Conclusions

Patients admitted for subacute in-hospital rehabilitation were able to anticipate their discharge health-related quality of life on the EQ-5D instrument with a moderate level of accuracy. This finding adds to the foundational empirical work supporting joint treatment decision making and patient-centered models of care during rehabilitation following acute illness or injury. Accurate patient expectations of the impact of treatment (or disease progression) on future health-related related quality of life is likely to allow patients and health professionals to successfully target interventions to priority areas where meaningful gains can be achieved. Accurate expectations may also help avoid despondency associated with falling short of unrealistic expectations or a lack of motivation associated with underestimating the potential for improvement in health-related quality of life.

## Competing interests

The authors declare they have no competing interests.

## Authors' contributions

SM contributed to research idea conception, data collection, data analysis and manuscript preparation, as well as manuscript review, appraisal and editing. TH contributed to research idea conception and manuscript review, appraisal and editing. Both authors read and approved the final manuscript.
